# Planning Your Route: Where to Start?

**DOI:** 10.1007/s42113-018-0018-0

**Published:** 2018-12-04

**Authors:** Lahari Sengupta, Radu Mariescu-Istodor, Pasi Fränti

**Affiliations:** Machine Learning, School of Computing, University of Eastern Finland, Joensuu, Finland

**Keywords:** Orienteering, Location-based game, Tour planning, Tour optimization, TSP, Human performance, O-Mopsi

## Abstract

Tour planning is an important part of location-based applications. A tour planner provides an optimized path through places of interests (targets) by minimizing the tour length or by applying some other constraints. It is usually formulated as a travelling salesman problem (TSP) or vehicle routing problem (VRP). In the present study, we focus on how to choose the best starting location in case of an open-loop TSP. We consider three different strategies for selecting the starting location and compare their effectiveness with regard to optimizing tour length. If all targets are visible, most humans tend to start on the convex hull or from the furthest point. However, there are also cases where not all targets are visible beforehand, and the only information given is the bounding box. An optimum tour then typically starts from the corner or the shorter side of the box. Humans also have a strong preference to start from a corner. A good strategy can result in the shortest tour, while a bad strategy can even add 20% to the total tour length.

## Introduction

The increasing popularity of sharing personal contents including photos, videos, and locations via social media has triggered an increasing interest in multiple recommendation systems. This interest has spawned an area of research that plays a vital role in building smart tourism. A survey by Borràs et al. (2014) reveals different web- and mobile-based tourism recommendation systems whereas Gavalas et al. (2014) focused mainly on comparing mobile-based recommendation systems. These studies consider two challenges: (1) how to collect the content from social media, i.e., the *places of interest* (POI) for tourism, and (2) how to plan tours between the collected POIs. In this work, we focus on the second challenge by studying human problem-solving skills when applied to tour planning.

Automatic recommendation systems aim at providing tours by considering context awareness, personalization, and suitability for a tourist trip. Yu and Chang (2009) and Lim et al. (2018) provided personalized tours based on the interest, need, and preference of the individual user. Majid et al. (2013) studied recommendation systems utilizing the *Flickr* image database. While the *TripPlanner* software (Chen et al. 2015) adds the users’ preferred venues iteratively to a candidate tour, *eCOMPASS* (Gavalas et al. 2015) favors public transit aiming to minimize the environmental impact. Both systems can be used for real-time tour planning. Keler and Mazimpaka (2016) provide safety routing by avoiding areas within a city that are considered dangerous.

Several studies have also focused on optimizing tours using explicit criteria. De Choudhury et al. (2010) recommended trips based on popularity within a restricted time budget. Gionis et al. (2014) provided tour recommendations based on fixed start and end points while accounting for a specific time or distance budget. Bolzoni et al. (2014) proposed clustering-based tour planning. Mor and Dalyot (2018) studied how to calculate distance-optimized walking tours using a bi-dimensional *nearest neighbor* (NN) algorithm based on geo-tagged photos from social media. Li et al. (2017) combined both travel time and ride comfort in their tour planner. All these approaches considered the travel planning as an *orienteering problem* (Vansteenwegen et al. 2011).

Classical orienteering explores the navigational skill of participants. *Mopsi orienteering* (*O-Mopsi*) is a mobile orienteering game (Fränti et al. 2017) where the targets are real-world objects such as POIs in a smart tourism system. Unlike classical orienteering, O-Mopsi does not have a pre-defined visiting order of the targets. Consequently, finding the optimal tour corresponds to solving an *open-loop travelling salesman problem*, which has been shown to be a non-deterministic polynomial-time (NP) hard problem (Papadimitriou 1977). This means that large-scale instances cannot be solved by a computer in a reasonable amount of time. However, small-scale instances can make a good puzzle for humans to solve. Other popular puzzle games that are computationally hard are *Sudoku* (Ercsey-Ravasz and Toroczkai 2012) and *Minesweeper* (Scott et al. 2011).

**Fig. 1:**
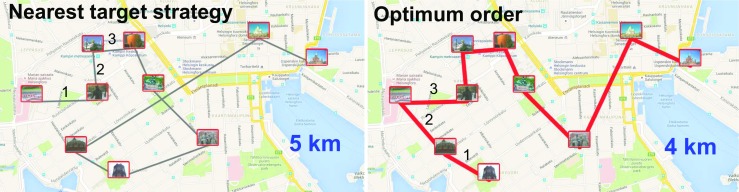
The tour length for a greedy nearest target strategy is 25% longer than the optimum tour length for the game named Helsinki downtown

In practice, O-Mopsi players rarely manage to find the optimum order but proceed by making heuristic choices. The most typical heuristic is to go to the nearest unvisited target until all targets have been visited. However, the effect of using this approach is demonstrated in Fig. 1. If a player starts at the leftmost target and follows this greedy *nearest target* (NT) strategy, he/she would end up travelling 1 km more than the optimum tour. Our experiments with O-Mopsi games revealed that the greedy strategy in combination with a random starting location can produce tours that are from 0.06% (best) to 109% (worst) longer than the optimum (median gap is 20%).

**Fig. 2:**
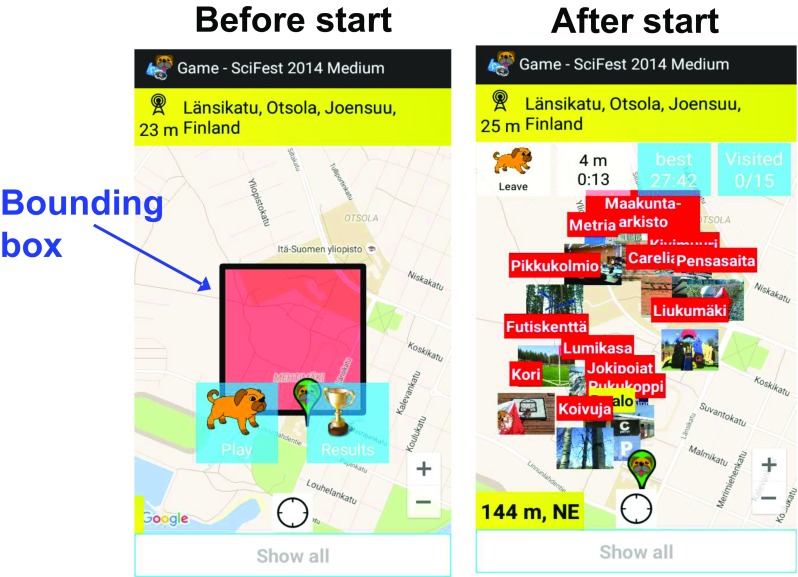
O-Mopsi screenshots taken before and after starting to play

Another challenge for O-Mopsi players is that before the game starts, they can only see the area (bounding box) containing the targets but their exact locations are not visible (Fig. 2). Therefore, they cannot plan their route beforehand, and after the game has started, any time spent on planning is added to the actual travelling time. This makes the planning even more challenging. An experienced player can do the planning while moving but for most players, it is a compromise of how much time to spend on planning and how much for the actual movement. This encourages players to develop different strategies for solving the order of visiting the targets fast.

**Fig. 3:**
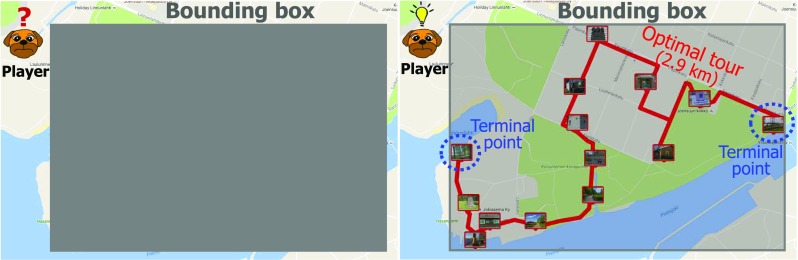
Challenge of the choosing starting position: situation before the playing starts (left) and the optimum order (right)

One important decision that players can make before starting the game is to select their starting location. Although the players do not know the actual locations of the targets, they can speculate. Firstly, it makes no sense to start the game while you are far away from the game area; secondly, players can make educated guesses about the target locations as the bounding box essentially reveals the partial locations of at least four targets: the longitudes of the left- and rightmost targets and the latitudes of the top- and bottommost targets; thirdly, the shape of the bounding box can provide hints about the layout of the optimum tour. The player can, therefore, move to the best strategic starting position before pressing the game’s start button (Fig. 3).

In this paper, we study different strategies for selecting the best starting point of an open-loop travelling salesman problem and study how human players perform in this. We statistically evaluate the goodness of various starting locations. We present three strategies to make the choice and compare their performances in terms of gap, aspect ratio, and how frequently they result in the optimum tour.

## Human Performance

Most research has studied human performance in solving the TSP with varying problem size and the number of points on the convex hull. Researchers have also focused on designing algorithms that best match human performance.

### Linear Relationship

Human skills in solving the TSP have been widely studied. MacGregor and Chu (2011) reported that humans can outperform simple TSP algorithms for relatively small TSP instances. Graham et al. (2000) showed that the time needed by a human to solve a TSP is linearly proportional to the size of the problem and that the gap to the optimal solution grows very slowly with the number of targets. Dry et al. (2006) made similar observations and found that the average time needed by a human to solve TSPs was linearly or near-linearly related to its size. Vickers et al. (2003a) showed that human performance worsened when more points were located on the convex hull.

### Modeling Human Behavior

Several researchers have tried to model the human capacity for problem-solving. MacGregor et al. (1999) compared three heuristics and found that the *convex hull* heuristic was the best fit for human approaches to solving the TSP. They suggested that people solve problems using a global-to-local perceptual process. According to this concept, they proposed an algorithm (MacGregor et al. 2000). Graham et al. (2000) found that none of the five algorithms they studied was an adequate model of the mental process involved in human TSP solving. Instead, they proposed a hierarchical algorithm, which is closer to the psychological process of the human problem solver. Pizlo et al. (2006) later refined this algorithm and showed that it produces solutions that came very close to those produced by humans.

Van Rooij et al. (2003) postulated the *crossing avoidance* hypothesis. They claimed that humans are intuitively aware that a tour with crossing trajectories is not optimal. Therefore, humans typically avoid crossing trajectories in their optimal tour planning for the TSP. The same authors also claimed that there was a lack of evidence to support the convex hull hypothesis of MacGregor et al. (1999, 2000). Nevertheless, MacGregor et al. (2004) re-stated that the convex hull hypothesis provides a stronger correlation with human performances than the crossing avoidance hypothesis.

In a detailed analysis of both the global-to-local and local-to-global approaches, Vickers et al. (2003a, 2003b) observed that humans typically prefer to solve a TSP through the local-to-global approach such as the *nearest neighbor* technique. Their results are also applicable to the open-loop scenario. However, they found no evidence that humans would prefer the convex hull approach in the open-loop case. Graham et al. (2000) pointed out that the applicability of the convex hull approach was limited to the closed-loop TSP and did not extend to the open-loop problem.

### Convex Hull

This heuristic constructs a convex hull of all unvisited points. At each step, the next point is chosen such that the path never crosses this convex hull. This process is repeated until all the points have been visited. Macgregor et al. (2006) concluded that the convex hull approach was closer to human performance than either of two other heuristics they examined (crossing avoidance and nearest neighbor). They only used rather simple heuristics yielding results that were inferior to results produced by humans. While path length captures an important aspect of the solution, it merely reflects the goodness of the algorithm and is not indicative of human behavior. Macgregor et al. (2006) also measured the similarity of the paths by counting how many arcs the solutions shared. It shows that the convex hull correlated better to human behavior than to the plain nearest neighbor heuristic. In addition, this heuristic is somewhat non-human and it would be surprising if humans were constructing convex hulls in their head while solving the problems. It would be more human-like to apply the nearest neighbor heuristic with some level of additional intelligence to avoid crossings and “dead ends.” Wiener et al. (2009) claimed that human performance is better than a pure nearest neighbor strategy. Therefore, the algorithm that correlates best with human behavior is still unknown.

In the following, we will merely focus on how to select the start point. Studies in this are very sparse in literature. Furthermore, players also need to plan based on the bounding box instead of the convex hull or nearest neighbor.

## Where to Start?

MacGregor (2012) studied the human tendency to select the starting point based on two strategies: *boundary* targets or *interior* targets of the convex hull shape of the set of targets. Results showed that humans preferred (71%) to start on a boundary. With respect to the shape of the bounding box, in our analysis, we, therefore, consider the three following strategies to choose the starting point:

- in the middle
- in a corner
- at the short side of the box

**Fig. 4:**
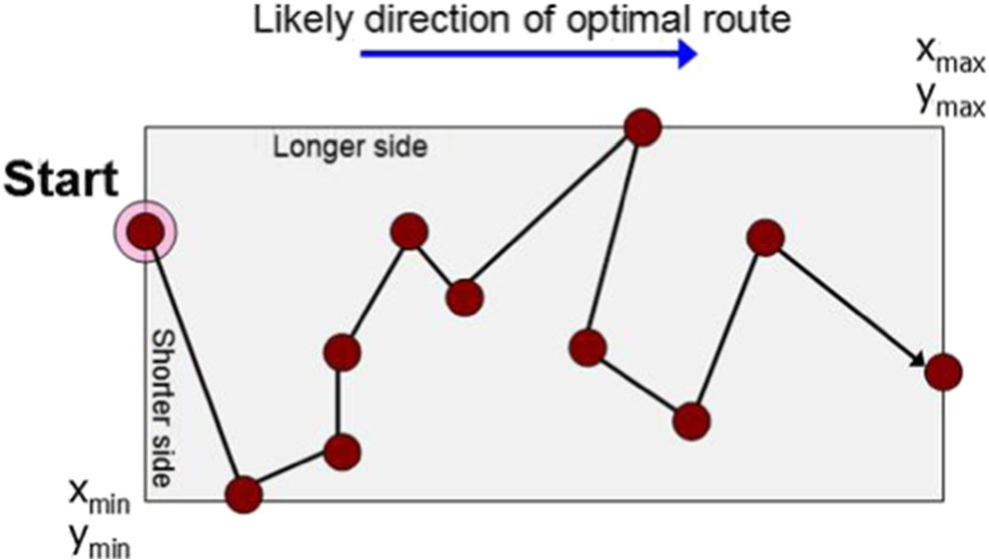
Most likely orientation of an optimum tour: starting and ending at the opposite (shorter) sides of the bounding box

Starting from the middle is the safest choice as it is very likely that there are some targets nearby. However, the optimum tour rarely starts from the center as it would form a spiral-shaped route. It is much more likely for the optimal route to start from a side (or corner) of the bounding box and finish at the opposite side (corner) (Fig. 4). Corners or sides of the bounding box are therefore expected to be a better starting point. Intuitively, it seems more likely that the optimum tour should also run along the direction of the long side of the bounding box than along the direction of the short side. This property leads to our third strategy.

**Fig. 5:**
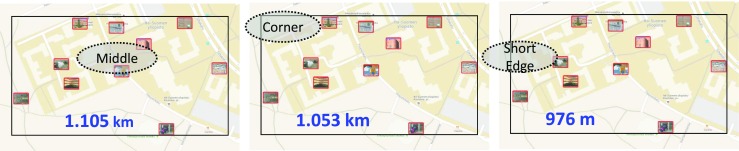
Effect of the three strategies on the tour length for “SciFest 2014 short” game (Fränti et al. 2017)

Figure 5 demonstrates the situation with “SciFest 2014 short” game with 10 targets where optimum tours are constructed from three fixed start points. By starting on a short side, the tour is shortened by 12% compared to starting in the middle and by 7% compared to starting from a corner.

### Game Area

**Fig. 6:**
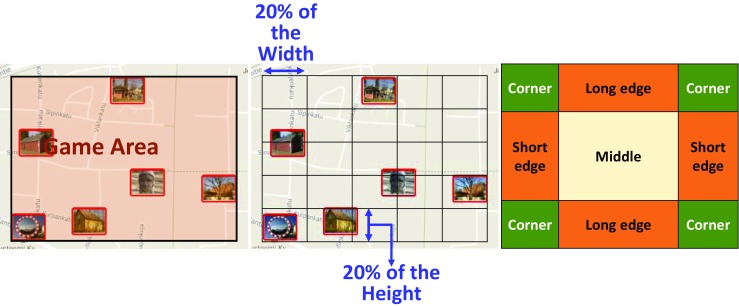
The game area is the bounding box, which is segmented by a grid into 25 cells named as a corner, long and short edge, and middle

We use the 147 O-Mopsi datasets^1^ (Fränti et al. 2017) to study the effect of choosing different starting points on route length. The *game area* is defined as the bounding box containing all targets of a game. We then divide this area into a regular 5 × 5 square grid. In accordance with the three starting point selection strategies, each grid cell is categorized as either a *middle*, *corner*, *long edge*, or *short edge* cell (Fig. 6).

If the *aspect ratio* (AR) (the ratio of game area width to height) is less than 1, the game area is rotated by 90° to ensure that the horizontal edge always corresponds to the long edge. AR is used as an additional indicator as the starting point of the optimum route is likely to lie on the short side if the game area is narrow (high AR values).

### Solving the Optimum Tour

**Fig. 7:**
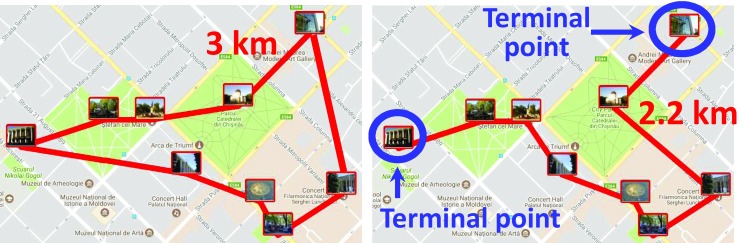
Solving the optimum order in the closed-loop case (left) and the open-loop case (right)

To solve the optimum route, we use the *Concorde* solver (Applegate et al. 2011) with two modifications. While the Concorde solver is designed for closed-loop cases, our scenarios consist of open-loop TSPs where the start and end points (*terminal points*) are different (Fig. 7). We remedy this by adding an equidistant (zero distance) phantom node to all targets as per Papadimitriou (1977). After solving the closed-loop solution, we remove this phantom node to obtain the corresponding open-loop solution. The two nodes connected to the phantom node are the terminal nodes for the open-loop problem. It is rather straightforward to show that this provides the optimum solution for the open-loop case, too.

**Fig. 8:**
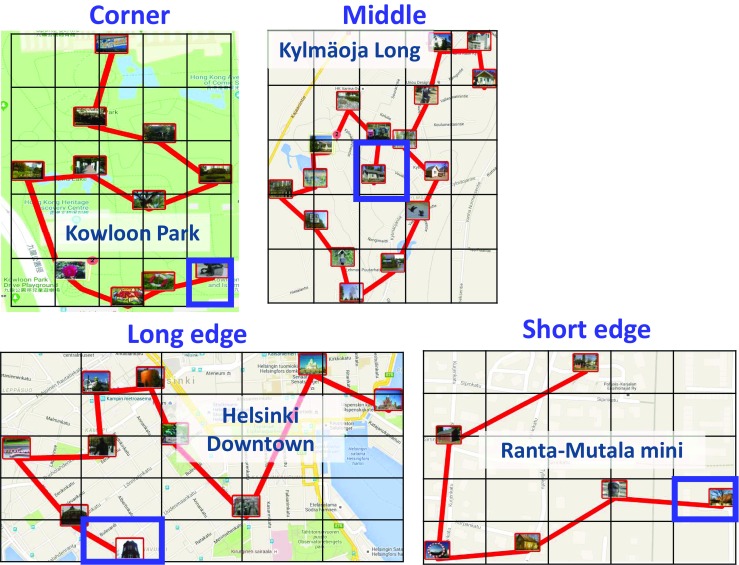
Examples of terminal points being in the corner, edge, and middle

**Fig. 9:**
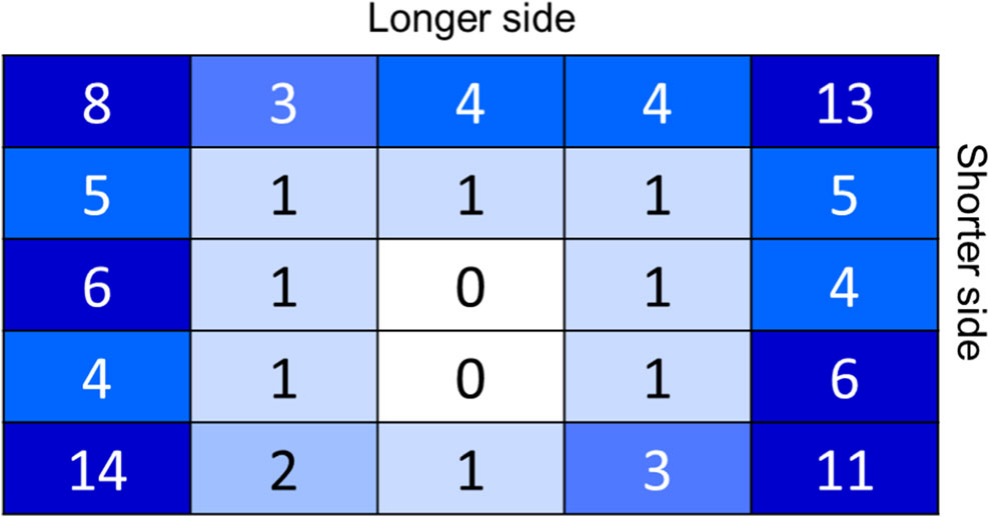
Probability (%) that the optimum terminal point is in the different grid locations

We find the terminal points of the optimum tours of O-Mopsi games. Figure 8 shows a few selected examples where the terminal points lie in the corner, along with the long and short edge, and in the middle. The probability for each cell to contain a terminal point of the optimum tour is given in Fig. 9. The observed (specific to our dataset) probabilities are compared against a priori probabilities (i.e., if the terminal points were randomly distributed) in Table 1. We find that corners are most likely to contain a terminal point (46%), whereas the short sides are almost twice as likely to contain a terminal point compared to the long sides (30% vs 17%). Clearly, middle cells are least likely to contain a terminal point (7%) although they represent the categories with the largest number of cells.

**Table 1:** Probability of a terminal point being located in a given cell

	**Cells**	**Probability**	
		**A priori**	**Observed**
Any corner	4	16%	46%
Any short side	6	24%	30%
Middle	9	36%	7%

**Fig. 10:**
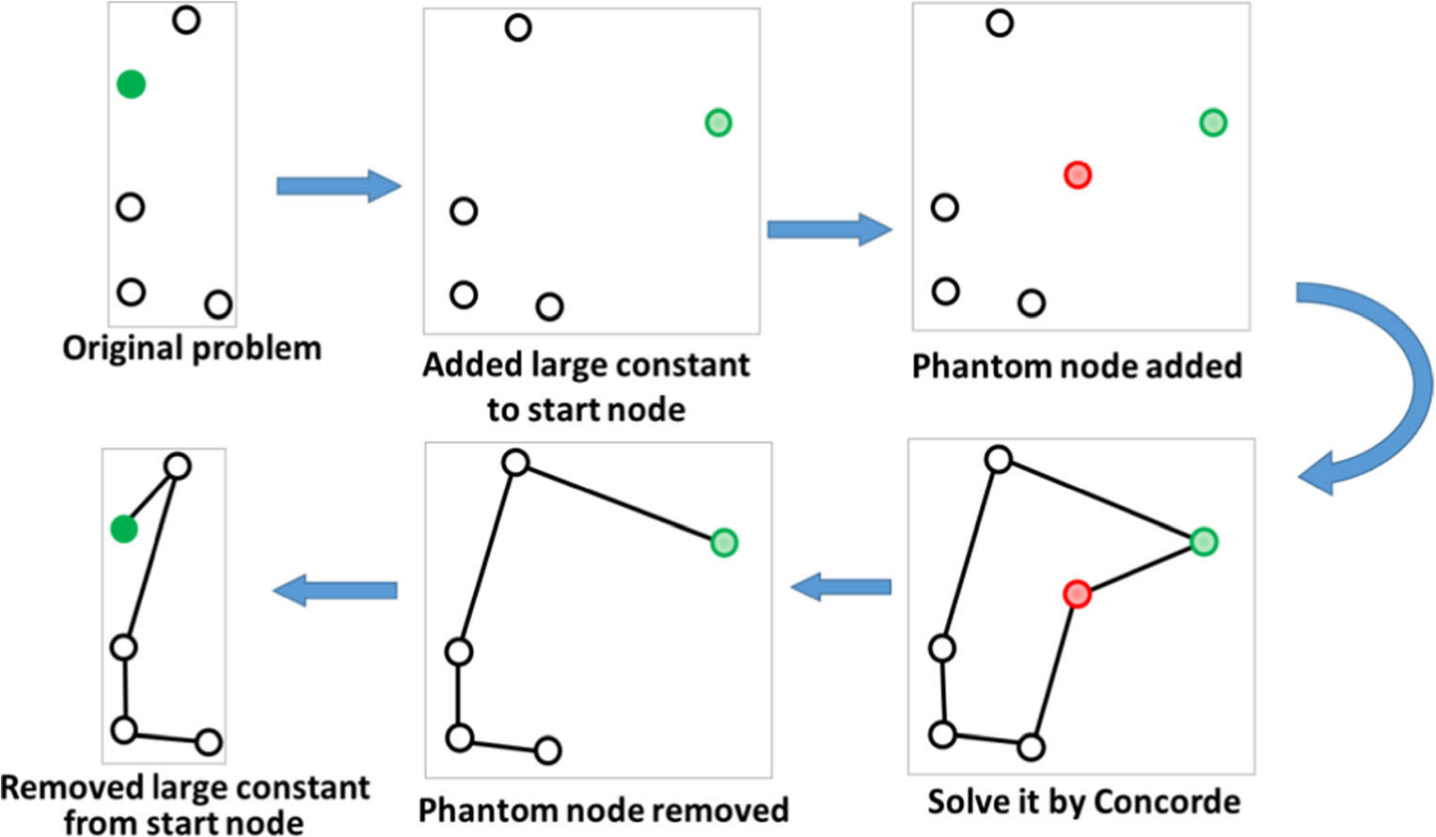
Finding the optimum order using Concorde with using the fixed starting point. The green dot is the player-selected start location, and red is the phantom node that is connected to the two terminal nodes

One can start playing from any point of the game area, which might not be a target, and it usually happens in practice. Thus, the second modification we applied to the standard Concorde approach is to create an additional node that represents a player’s start location. This allows us to calculate the optimum order from any location, not only starting from one of the targets. To force the solver to use this additional node as one of the terminal points, we add a large constant (twice the longest pairwise distance between any two targets) to all distances from this location, except to the distance to the phantom node which remains zero. This forces the solver to use these large distances exactly once and connect it to the phantom node, thereby ensuring that it is a terminal node. The final result is the optimum solution with the fixed start point (Fig. 10).

**Fig. 11:**
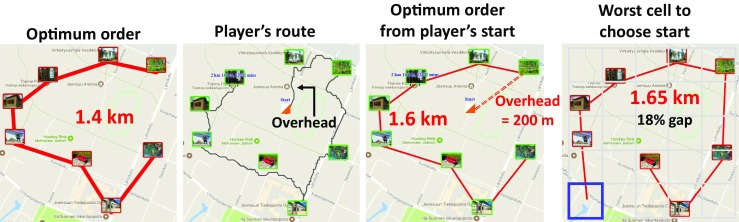
Effect of the starting location chosen by the player. The reference result of the optimum solution is also given when starting from the player start location. The worst possible starting location is also shown (right)

We then use the Concorde solver to evaluate different start locations as follows. We consider the center of each 5 × 5 grid cell as a potential starting location. This gives 25 different choices: 9 in the middle, 4 at corners, 6 at the long edge, and 6 at the short edge. By finding the optimum tour for each of these 25 starting locations and comparing the result against overall optimum, we can determine the *best* starting grid cell that yields the shortest tour for a given game. Similarly, the starting point grid cell that results in the longest tour is labeled the *worst* (Fig. 11).

### Effect of the Road Network

**Fig. 12:**
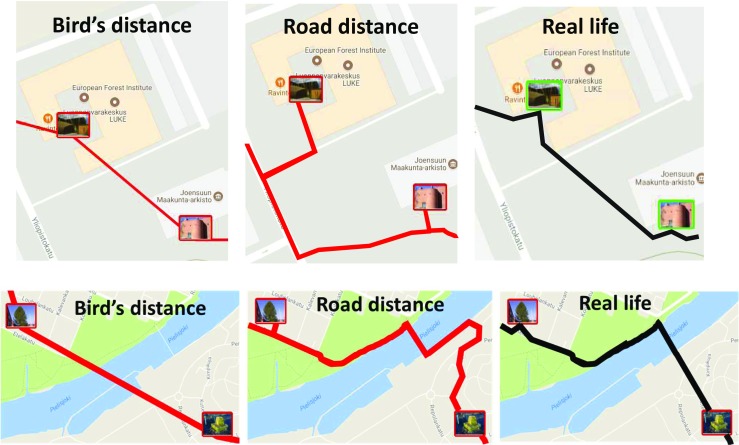
Cases when bird’s distance gives more realistic estimation (above), and when road network gives more realistic estimation (below) of the distance travelled in real life

Using bird’s (haversine) distance to measure the distance between targets underestimates the actual walking distances along urban roads and footpaths. Alternatives like *OpenStreetMap* (OSM) could be used to compute the shortest path while taking street layout into account and obtain more realistic distance estimates, particularly in urban areas. This may not be applicable to situations where games are set exclusively in parks or campus areas where players can take shortcuts that do not correspond to the road network (Fig. 12). In practice, neither approach is perfect, and both have a strong correlation to players’ preferences and choices. Nevertheless, the bird’s distance correlates slightly higher (0.95) with distances traversed by players than distances obtained using the road network (0.93). For these reasons, we use the bird’s distance in this study.

## Computer Performance

For our study, we use the O-Mopsi dataset (Fränti et al. 2017) as summarized in Table 2. The games have been manually created all over the world. Most games are set in Finland, predominantly in the Joensuu area, with an average game length of 3.5 km and an average number of targets of 12.

**Table 2:** Datasets used in this study

**Dataset**	**Type**	**Distance**	**Instances**				**Sizes**
O-Mopsi^a^	Open loop	Haversine	Total	Low AR < 0.8	Medium AR = 0.8–1.2	High AR > 1.2	4–27
			147	43	49	55	

^a^ http://cs.uef.fi/o-mopsi/datasets/

### Location of the Terminal Points

From Table 1, we already know that corners are the most probable zone to have terminal points. Now, we want to have an insight into this probability depending on the variation of the aspect ratios. From Table 3, we observe that for games with high AR or low AR, corners have the highest probability, followed by short edges, long edges, and lastly middle cells. However, unlike high and low AR games, in the case of the square-shaped game area (AR ≈ 1), there is no significant difference in probabilities between edges and middle cells.

**Table 3:** Probabilities of strategies for different game area aspect ratios (AR)

	**Low** **AR < 0.8 (%)**	**Medium** **AR = 0.8–1.2 (%)**	**High** **AR > 1.2 (%)**
Corner	48	46	45
Short edge	31	21	37
Long edge	17	23	16
Middle	4	10	2

**Fig. 13:**
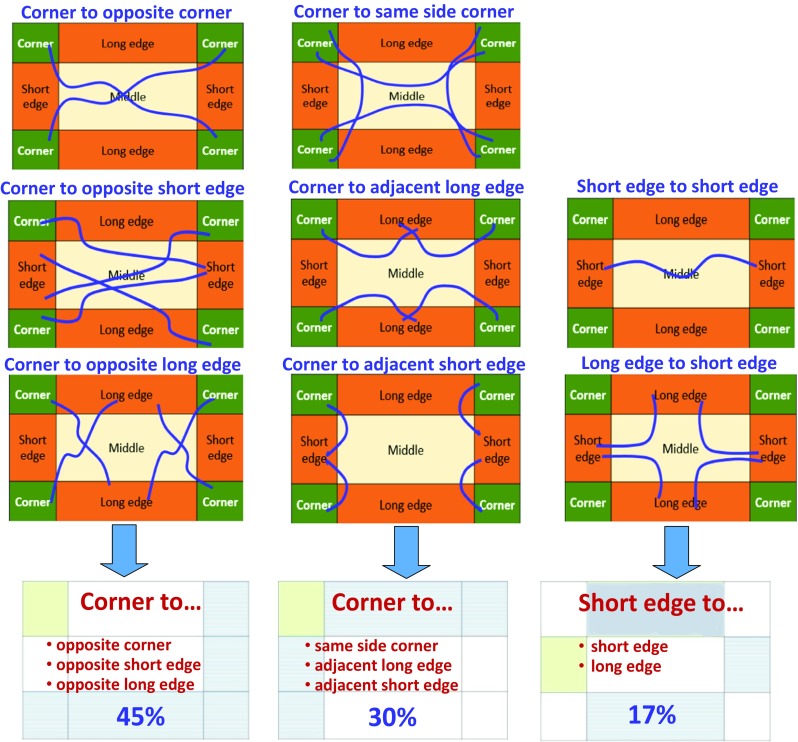
Extended classification (above) of the start point locations, and their share among all O-Mopsi games (below). Only the most common patterns are shown

In order to examine the layout of the optimum tour through the grid cells, we extend the classification of the layout of the optimum tour as shown in Fig. 13. In this figure, only those patterns are shown that are found in the dataset. For instance, pattern like middle-to-middle, for instance, never occurs in any of the games. Other rare patterns include long edge to middle (1%), short edge to middle (4%), and corner to middle (5%). While almost half the games have an optimum solution with a corner to opposite side/to opposite corner pattern (45%), the overwhelming majority (92%) spans between corner and edge terminal points.

Table 4 illustrates that the optimum tour usually extends from one corner to the opposite edge or corner for games with high and low AR. Hence, for these games, the optimum tour is often like the example in Fig. 4. For more square games, no clear trend emerges; all types of orientation are almost equally probable. So, by seeing the square game area, it is more difficult for the player to guess the probable optimum tour orientation, whereas for low AR or high AR game area, there are more hints to make an educated guess.

**Table 4:** Game expansion with varying widths of game areas

	**Low** **AR < 0.8 (%)**	**Medium** **AR = 0.8–1.2 (%)**	**High** **AR > 1.2 (%)**
Corner to opposite corner/edges	58	29	50
Corner to same corner/adjacent edges	12	29	20
Short edge to other edges	23	20	25
Others	7	22	5

### Player’s Start Position

Here, we include an extra location to the targets of a game to simulate a player’s playing. We consider each 25 grid cells as a potential starting location for a player, and with respect to the tour length, we find the best one among them. Overall optimum tour tends to have terminal points at the corners. It is therefore expected that the best start position among the 25 grid cells is very likely to be there.

By calculating the probabilities of grid cells to be the *worst* starting position, we observe that corners are also the most likely (45%) the worst start location. Thus, it is possible that for a particular game layout one corner may be the best starting location while another corner may be the worst. If a player is unlucky, then he/she could choose the worst corner. Evidently, corners are risky locations to start. Apart from corners, also the long edge (32%) and middle (17%) are likely to contain the worst starting position, while the short edge is the least likely (6%) to contain the worst starting position.

In order to examine how bad the worst location is, we calculate the gap (%). On average, the worst starting locations resulted in a 16% longer tour than the best start with no significant dependence on AR (18% for low, 14% for medium, and 16% for high AR). However, medium AR games have the lowest gap value. Therefore, for square games, the difference between the worst and the best start is the least noticeable, which makes these games tougher to predict the best start.

In practice, players in real life do not consider such sophisticated start point selection strategies. Instead, they merely start at a random location—often even outside the game area. However, this random strategy is very unlikely to result in an optimum tour (Table 5) and we can conclude that the starting point selection strategy has a significant effect on finding the best solution. While starting from a corner has a 32% chance of success, a randomly selected starting location only results in an optimum route in 3% of cases. However, the gap is < 10% in all cases and does not vary as much as expected. The best overall choice is to start on a short side (6% average gap) while the worst place to start is the middle (9% average gap).

**Table 5:** Comparing different starting point selection strategies

	**Probability to find best (%)**	**Gap to best (%)**
Random	3	8
Middle	9	9
Any corner	32	7
Any short edge	23	6

## Human Results

### Test Setup

Experimental data was collected from volunteers (students) participating in the *Design and Analysis of Algorithms* course at the School of Computing at the University of Eastern Finland in September 2018 (http://cs.uef.fi/pages/franti/asa). Two types of game setup were designed: *visible* (targets are visible) and *blind* (only bounding box is visible). In the visible setup, the students were given 90 instances (one at a time) and instructed to select the point where they thought the optimal tour would start. From this starting location, *Concorde solver* then computed the optimal tour. We then calculated the gap between the optimum tour with the student-selected fixed starting location and the overall optimum tour for the given target set. In the blind setup, the students saw only the bounding box but not the individual target locations. In our analyses, we include results from students who participated in both tasks (visible and blind) and had completed more than 60 tasks overall.

**Fig. 14:**
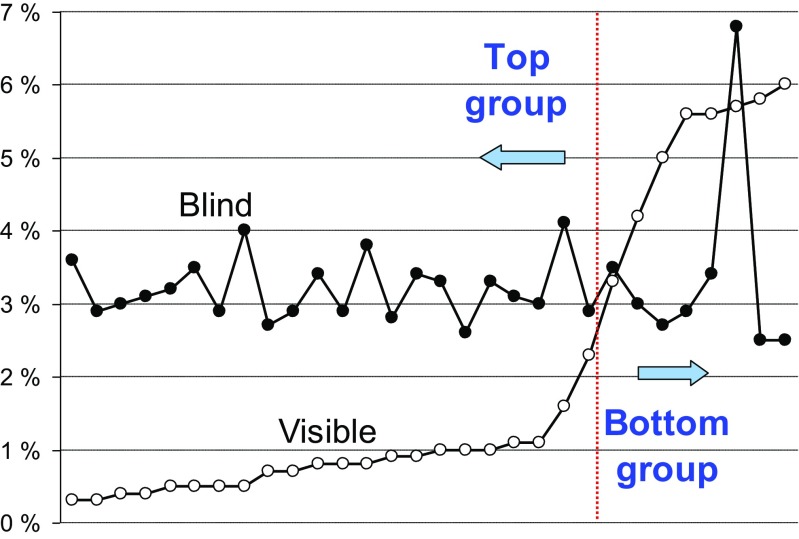
Human performance (gap) in the selection of a start point

We sort the results according to performance (average gap) and subdivide them into two subgroups: *top* and *bottom* (Table 6, Fig. 14). From this result, we observe a clear jump in performance around the 2–3% gap. Several other indicators also suggest that the bottom group (8 students) might not have understood the problem well. The strongest evidence is that they performed better in the blind task compared to the visible task.

**Table 6:** Results of the human experiments both for the visible and for the blind tasks. Rounds means tasks completed. Solved means number of times optimal solution found. Gap means average gap of the tour length and the optimal tour. Hull means number of times started on the convex hull. Furthest means number of times started from the furthest point. Corner means number of times started from the corner

**Top group**								
**Visible**					**Blind**			
**Rounds**	**Solved**	**Gap**	**Hull**	**Furthest**	**Rounds**	**Solved**	**Gap**	**Corner**
90	78	0.3%	100%	58%	86	31	3.6%	93%
90	77	0.3%	99%	51%	90	35	2.9%	99%
90	75	0.4%	97%	42%	90	40	3.0%	100%
90	77	0.4%	98%	39%	90	37	3.1%	97%
65	53	0.5%	100%	55%	89	35	3.2%	95%
90	74	0.5%	99%	49%	90	33	3.5%	95%
90	76	0.5%	98%	42%	90	39	2.9%	98%
89	70	0.5%	93%	35%	90	27	4.0%	56%
90	78	0.7%	93%	42%	90	42	2.7%	100%
90	67	0.7%	98%	40%	90	38	2.9%	91%
90	63	0.8%	90%	29%	90	30	3.4%	83%
90	68	0.8%	99%	37%	90	43	2.9%	98%
90	66	0.8%	98%	49%	90	23	3.8%	88%
90	63	0.9%	97%	44%	90	41	2.8%	93%
89	64	0.9%	100%	55%	90	36	3.4%	79%
90	57	1.0%	99%	34%	90	37	3.3%	100%
90	62	1.0%	92%	31%	90	40	2.6%	100%
90	64	1.0%	91%	44%	90	37	3.3%	99%
90	58	1.1%	99%	44%	90	37	3.1%	83%
90	64	1.1%	91%	44%	90	41	3.0%	98%
90	58	1.6%	96%	20%	90	28	4.1%	74%
90	55	2.3%	90%	36%	90	32	2.9%	100%
Average		0.8%	96%	42%	Average		3.2%	92%
Bottom group								
Visible					Blind			
Rounds	Solved	Gap	Hull	Furthest	Rounds	Solved	Gap	Corner
78	24	3.3%	59%	15%	90	34	3.5%	82%
90	17	4.2%	37%	11%	90	43	3.0%	100%
87	14	5.0%	46%	10%	90	38	2.7%	100%
90	14	5.6%	29%	7%	90	42	2.9%	92%
90	11	5.6%	29%	7%	90	37	3.4%	93%
79	9	5.7%	32%	5%	90	6	6.8%	3%
90	6	5.8%	24%	1%	90	44	2.5%	100%
90	4	6.0%	18%	2%	90	39	2.5%	99%
Average		5.2%	34%	7%	Average		3.4%	84%

### Visible Setup

Most students achieved an average gap of less than 1%. To further investigate human performance, we calculate two additional parameters for each game: (i) *convex hull* and (ii) the *furthest point* from the center. We label each point whether it is *on* the convex hull or not. MacGregor (2012) suggested that humans may tend to start from a point on the convex hull. Our results confirm this. Starting on the convex hull also strongly correlates with performance. The top group almost always selected starting points on the convex hull (96% of times), while the bottom group used this strategy less frequently (34%).

Students strongly preferred to choose the furthest point in the game layout as the starting point. The top group chose it for 42% of cases. Although only a few students could explicitly describe their selection strategy when asked to do so, most of them told that they chose “most obvious outlier” point as the starting location. One student described his strategy was choosing the “leftmost or rightmost point whichever was further.”

### Blind Setup

All students who were in the top group for the visible setup performed significantly worse in the blind setup. Furthermore, there is almost no difference in performance between players and no correlation between the visible and blind performances. The average gaps are 3.2% (top group) and 3.4% (bottom group) which is only slightly better than if the start point was chosen randomly (4.0%). This indicates that the skills required for the blind setup have very little in common with the skills required for the visible setup. Either the skill sets for both variants are completely different, or the blind setup requires more time to learn the necessary skill.

Here, most players found the computer-preferred corner point strategy. Top group students applied the corner strategy in 92% of times. The results also show a clear correlation between performance and the corner point strategy. Those players (17) who selected a corner point > 95% times achieved an average gap of 2.9% whereas the rest (13) had an average gap of 3.7%. None of the players seemed to use the short edge or any other strategy. One player chose the middle point strategy in 91% of games which resulted in a clear outlier (6.8% gap).

There was no observable correlation between the start point strategy and the game area aspect ratio.

In conclusion, human solvers were generally unable to discover any sophisticated strategy with such short experience in the game. Our expert players (the three authors) used this short edge strategy slightly more often (35%) but still relied mostly on the corner point strategy (62%) despite knowing these strategies beforehand.

### Size of Instances

**Fig. 15:**
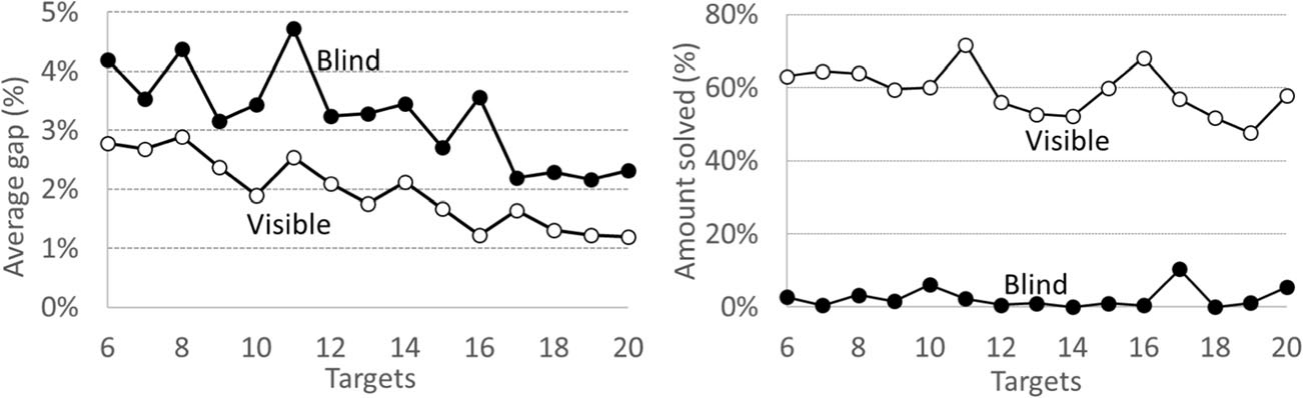
Dependency of the human performance on the instance size (targets)

Graham et al. (2000) suggested that human performance decreases only slightly when the number of targets increases. Our results in Fig. 15 are seemingly contradictory; with more points, the gap reduces. This could be explained by arguing that as the number of targets increases, the less important the choice of the starting point becomes. In our case, the human role was limited to select only the starting location while the computer solved the rest. So contrary to solving the entire TSP, this factor reduces the gap. However, in terms of success rate, finding the optimal result (0% gap) becomes more difficult when more points are added. In this sense, our results are in line with the previous findings in the literature.

Vickers et al. (2003a) showed that humans performed worse when the game had more points on the convex hull. We tested this hypothesis as well by dividing the games into two groups: one with a high number of points on the convex hull and one with a small number. We did not find any significant differences between both groups.

### Correlation to Study Performance

Finally, we compare human performance against their grades in the *Design and Analysis of Algorithms* course. Students were divided into two groups (high and low) according to their exam score. We tested two alternative hypotheses. Our primary hypothesis is that those who performed well in the course would also perform well in the TSP problem-solving.

**Fig. 16:**
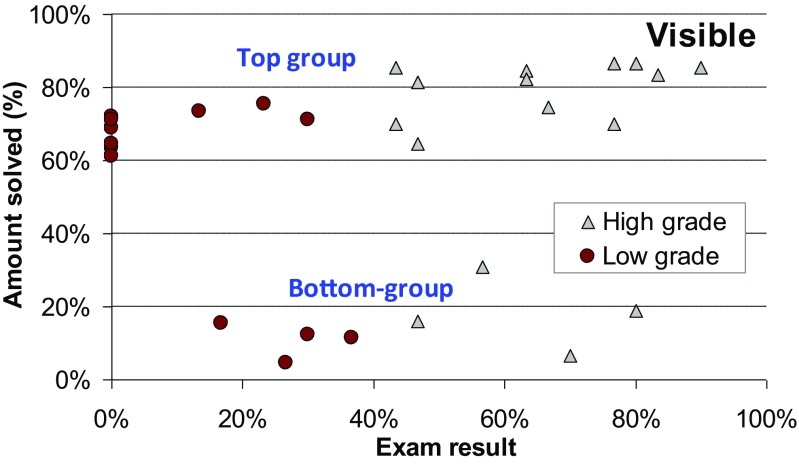
Correlation between human performance and their course grades

We count the number of games solved (in percentage) by each student to measure their TSP solving skill. The results in Fig. 16 show that there is a relatively low correlation with the grade and the TSP performance. There are high grade students both in the top group and in the bottom group, and surprisingly, all drop-outs (0% exam score) are in the top group. If we ignore the bottom group, we can see that the course performance still has significant predicting power. Top eight among the students who solved games achieved higher grades in the course exam as well.

**Fig. 17:**
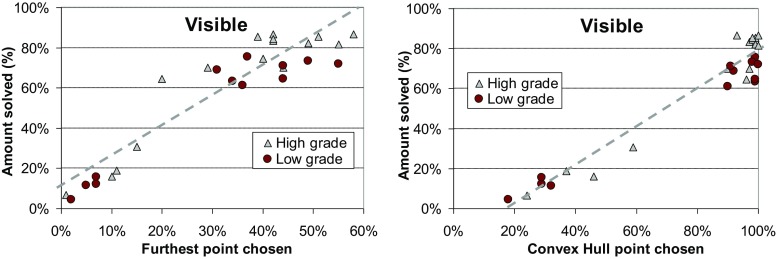
Correlation between human performance and their strategies

Students preferred points on the convex hull or the furthest point as the starting location. Next, we check how much these strategies matter. From Fig. 17, we find that the more often a student used (knowingly or sub-consciously) the furthest point and the convex hull strategy, the better was his/her problem-solving performance. At first sight, the course grade seems to have almost no effect, but again, if we consider only the top group, the course performance becomes obvious. Using the starting point selection strategy has been the most crucial for determining a student’s group, whether it is a top or a bottom. However, within the top group, the course performance has the strongest predicting power. Table 7 summarizes the correlation ratios between the three factors (furthest point, convex hull, course performance) with the problem-solving performance.

**Table 7:** Pearson correlation ratios between different factors with the amount of games solved

**Affecting factor**	**All**	**Top group**
Convex hull	**0.97**	0.38
Furthest point	**0.93**	0.53
Course performance	0.11	**0.72**

**Fig. 18:**
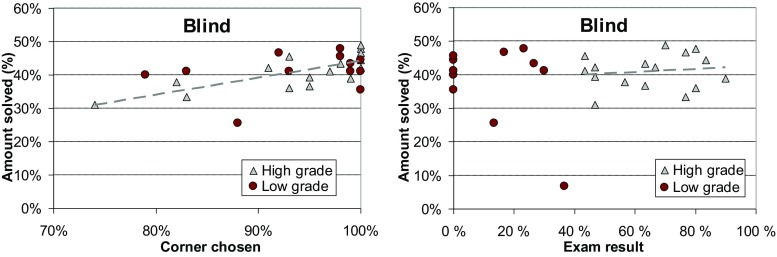
Correlation of human performance with corner strategy and exam score

For the blind variant, the most popular strategy was to choose any corner. From results, we find out that students choosing more corner points perform better (see Fig. 18). However, the correlation between the course scores and the TSP performance is not high.

## Conclusions

We have studied different strategies for selecting a start point for solving the open-loop travelling salesman problem. The results showed that games have almost equal chance of having the best starting point at any grid location, except corners, which were most often the best choice. At the same time, corners can also be the worst choice, which makes it also the riskiest choice. Most human players in our trial used solely the corner point strategy although the short edge strategy would have been a slightly better choice.

With visible targets, the choice of human players clearly correlated with the starting point being on the convex hull or being the furthest from the center although a few were able to formulate and particularly argument to justify their choices. When increasing the number of targets, the player performance started to slightly degrade when measured by how many times the optimal solution was found. However, using the gap as the sole measure of success can be misleading. In our case, it would falsely imply the games become easier with the increasing number of targets, but this is clearly not the case. The average gap merely shows that the performance difference becomes less significant when the problem size increases.

## Footnotes

1
http://cs.uef.fi/o-mopsi/datasets/
